# Arsenical Confectionery

**Published:** 1853-05

**Authors:** 


Mr. Editor,—On the 21st of February last, I delivered a lady of a male child which weighed eighteen pounds and a fraction. The child had been dead about three days. The labor was instrumental. I merely mention this case for its novelty. The lady is very large and has borne several children. Yours, J. M. MEYER, M. D.,

—*Transylvania Med. Journal.* Dansville, Ky.

